# PI3K activation is enhanced by FOXM1D binding to p110 and p85 subunits

**DOI:** 10.1038/s41392-020-00218-3

**Published:** 2020-06-30

**Authors:** Qi Wang, Pingzhao Zhang, Wei Zhang, Xin Zhang, Jianfeng Chen, Peipei Ding, Luying Li, Xinyue Lv, Ling Li, Weiguo Hu

**Affiliations:** Fudan University Shanghai Cancer Center and Institutes of Biomedical Sciences, Shanghai Medical College, Fudan University, Shanghai, 200032 China

**Keywords:** Oncogenes, Cell biology

**Dear Editor,**

Class I PI3Ks play a central role in cancer progression via its downstream signaling nodes (GSK3, FOXO, mTORC1, etc.).^1^ Under physiological conditions, the catalytic p110 subunit is stabilized and inhibited by the regulatory p85 subunit in the cytoplasm.^1,2^ In cancer cells, PI3K activity is aberrantly regulated mostly through excessive upstream growth signals, disinhibition of p110 by genetic alteration of PI3K genes, and deficiency of the phosphatase PTEN.^1^ Protein interactions between PI3K and overexpressed oncoproteins such as Ras also account for the activation of PI3K in cancer cells.^1^ Abnormally activated PI3K/AKT pathway is a popular drug target for cancer therapy; therefore, the mechanisms underlying the regulation of PI3K activity need the elaborate exploration.

Forkhead box M1 (FOXM1) was initially shown to sustain cell proliferation by regulating cell cycle progression.^3^ Four FOXM1 isoforms, FOXM1A/B/C/D, have been identified due to alternative splicing; FOXM1B and FOXM1C are located predominantly in the nucleus as transcription factors, while FOXM1A and FOXM1D are located exclusively in the cytoplasm.^4^ FOXM1 (which generally refers to FOXM1B and sometimes FOXM1C) is intimately involved in tumor initiation, proliferation, invasion and metastasis.^3^ FOXM1D was highly expressed in late-stage colorectal cancer with liver metastasis and found that it promotes epithelial–mesenchymal transition (EMT) by interacting with and activating ROCKs.^4^ Conditional deletion of FOXM1 in the liver rendered mice resistant to hepatocellular carcinoma development; Nevertheless, overexpression of FOXM1B displayed only negligible effects on hepatocellular carcinoma progression.^3^ The discrepancy suggests that in addition to FOXM1B, other isoforms of FOXM1 may also play important roles in cancer development.

Therefore, we identified the FOXM1D binding partners using co-immunoprecipitation (co-IP) and liquid chromatography–mass spectrometry. The result showed that p110α was a FOXM1D-interacting protein in FOXM1D-overexpressing HeLa cells (Fig. 1a, Supplementary Tables 1 and 2). Using co-IP (Fig. 1b) and immunocytochemistry (ICC) (Fig. 1c, Supplementary Fig. 1a, b, Supplementary Fig. 2) assays, we further confirmed and revealed the physical interaction of FOXM1D with p110α and other class IA catalytic subunits (p110β and p110δ). In addition, reverse co-IP (Supplementary Fig. 3a) and GST pull down (Supplementary Fig. 3b) assays further verified the interaction between FOXM1D and p110β. To map the precise binding region between FOXM1D and p110β, we generated the truncated mutants of FOXM1D and p110β to perform a co-IP assay (Fig. 1d, e, Supplementary Fig. 3c, d). The results demonstrated that FOXM1D directly interacts with the catalytic domain of p110β subunit via the exon VIII coding region. Further, the results of ICC assays also indicated that the catalytic domain of p110α/δ co-localized with FOXM1D (Supplementary Fig. 3e).

**Fig. 1:**
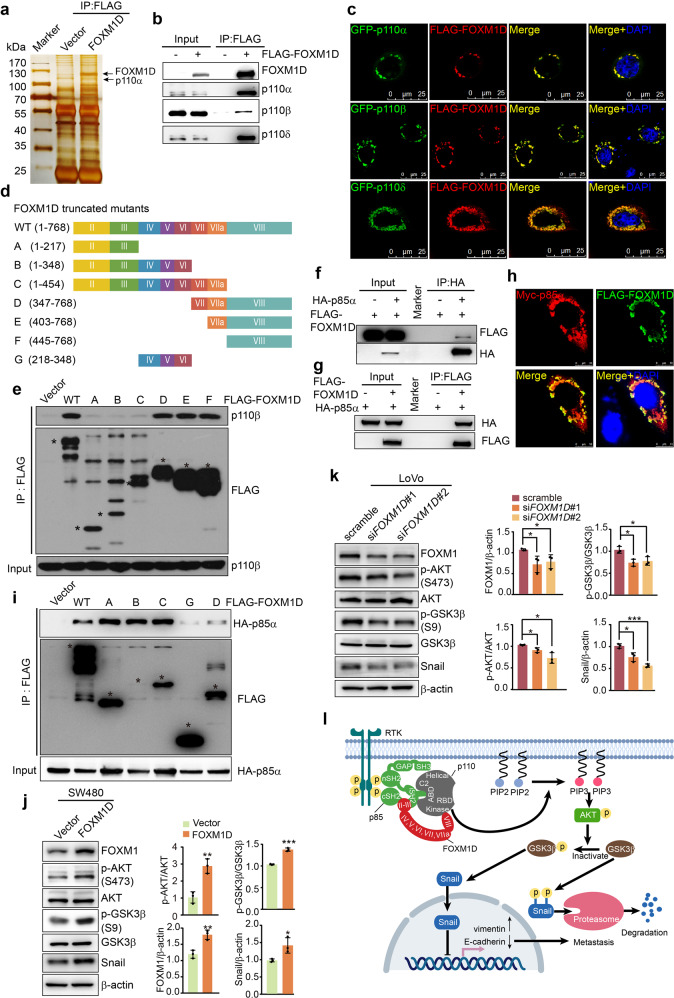
FOXM1D directly interacts with p110 and p85 subunits of PI3K and regulates PI3K activity. **a** The co-IP and silver staining assays to identify the FOXM1D binding proteins in the lysates from HeLa cells expressing FLAG-FOXM1D. **b, c** The co-IP (**b**) and ICC (**c**) assays to determine the interaction between FOXM1D and p110α/β/δ. Scale bar, 25 μm (**c**). **d, e** Identification of the mutual binding sites of FOXM1D and p110β. Co-IP assays were performed of the lysates from 293FT cells expressing FLAG-FOXM1D or FLAG-tagged truncated mutants of FOXM1D (**d**) using a FLAG antibody (**e**). The obtained samples were detected by IB using FLAG and p110β antibodies (**e**). **f**–**h** The co-IP (**f, g**) and ICC (**h**) assays to determine the interaction between FOXM1D and p85α. Scale bar, 10 μm (**h**). **i** Identification of the mutual binding sites of FOXM1D and p85α. Co-IP assays were performed of extracts from the 293FT cells expressing FLAG-FOXM1D or FLAG-tagged truncated mutants of FOXM1D (**d**) and HA-p85α-expressing plasmids using a FLAG antibody (**i**). The obtained samples were detected by IB using FLAG and HA antibodies (**i**). **j, k** The immunoblotting assays to determine the activation of PI3K/AKT/GSK3β/Snail signaling axis in SW480 cells with ectopic FOXM1D expression (**j**) and in LoVo cells with knocked-down FOXM1D expression (**k**). Error bars: mean ± SD (*n* = 3); Student’s *t*-test; *P < 0.05, **P < 0.01; ***P < 0.001. **l** The working model. FOXM1D binds to p85 in its junction region between iSH2 and cSH2 via its exon II- and III-encoding region, while binds to p110 kinase domain via its exon VIII-encoding region; therefore, the intermediary FOXM1D generates an effect of steric hindrance between p110 and p85, thus relieving the inhibitory effect of p85 on p110 kinase activity and enhancing the PI3K/AKT activation upon upstream signaling stimulation, which leads to increased phosphorylated GSK3β, stabilized Snail and accumulated nuclear Snail, contributing to the transcriptional repression of E-cadherin and elevation of vimentin, and cancer metastasis. *GF* growth factor, *RTK* receptor tyrosine kinase

To delicately execute their cellular functions, the class IA PI3K catalytic subunits p110α/β/δ form a heterodimer with the regulatory subunit p85. The results of reciprocal co-IP (Fig. 1f, g), ICC (Fig. 1h, Supplementary Fig. 4a, b) and GST pull down (Supplementary Fig. 4c) assays showed that FOXM1D concurrently interacted with p85α. In addition, the N-terminal region coded by FOXM1D exons II and III and the junction region between the iSH2 and cSH2 domains of p85α were identified to contribute to the interaction between FOXM1D and p85α (Fig. 1i, Supplementary Fig. 4d, e).

To further confirm that FOXM1D simultaneously binds to p110 and p85, we detected their co-localization using quadruple ICC assay. The results indicated that FOXM1D, p110β and p85α could simultaneously interact predominantly in the cytoplasm (Supplementary Fig. 4f). Next, we employed gel chromatography to detect the existence of this heterotrimeric complex. The predicted molecular weight of this complex is approximately 291 kDa, which comprises one molecule of FOXM1D (87 kDa), p85 (84 kDa), and p110 (~120 kDa). We observed that FOXM1D, p110α/β/δ and p85 appeared concurrently between 158 kDa and 440 kDa in the extracts of FOXM1D-overexpressing HeLa cells (Supplementary Fig. 4g), suggesting the probability of physiological existence of this heterotrimer.

Due to the high association of FOXM1D with metastasis,^4^ we next ascertained the effect of FOXM1D interaction with PI3K on the downstream signaling AKT/GSK3β/Snail.^5^ Compared to the control cells, SW480 cells with ectopic FOXM1D expression exhibited markedly enhanced AKT phosphorylation (Fig. 1j, Supplementary Fig. 5a), while FOXM1D-insufficient LoVo (Fig. 1k, Supplementary Fig. 5b) and HCT116 cells (Supplementary Figs. 5b and 6a) showed significantly attenuated AKT activity. PI3K/AKT signaling can be activated by extracellular signaling molecules such EGF by binding to EGFR.^1^ We observed that ectopic FOXM1D expression strongly enhanced AKT phosphorylation in a dose- (Supplementary Fig. 6b) and time-dependent manner (Supplementary Fig. 6c) in SW480 cells after EGF stimulation. Moreover, the activation of AKT could be dramatically suppressed by the pan-class I PI3K inhibitor BKM120 (Supplementary Fig. 6d) or p110β/δ specific inhibitor IPI145 (Supplementary Fig. 6e). Via the potentiated PI3K/AKT signaling, ectopic expression of FOXM1D also inactivated GSK3β via phosphorylation and subsequently increased the Snail level in SW480 cells (Fig. 1j). While FOXM1D insufficiency reduced the Snail level by compromising AKT activation and GSK3β phosphorylation in LoVo cells (Fig. 1k).

Given that Snail is a master regulator of EMT and FOXM1D potently promotes EMT in vitro and metastasis in vivo,^4^ we further examined the migratory abilities and EMT marker levels after altering FOXM1D expression. We found that the migratory ability was notably enhanced in FOXM1D-overexpressing SW480 cells, and potently suppressed in FOXM1D- insufficient LoVo cells in transwell assays (Supplementary Fig. 7a, b). Further, we observed that ectopic expression of FOXM1D reduced E-cadherin and increased vimentin expression in SW480 cells (Supplementary Fig. 7c), while FOXM1D insufficiency displayed the opposite effect in LoVo cells (Supplementary Fig. 7d). As expected, administration of BKM120 weakened the effect of ectopic FOXM1D expression on increasing the Snail level in SW480 by inhibiting the AKT activation and the resultant GSK3β phosphorylation (Supplementary Fig. 7e).

Snail phosphorylation by GSK3β accounts for its degradation by β-Trcp-mediated ubiquitination and its subcellular localization.^5^ We investigated whether this mechanism regulates the level and distribution of Snail in FOXM1D case. The transcriptional level of *SNAIL* remained unchanged regardless of the ectopic FOXM1D expression in SW480 cells and the FOXM1D insufficiency in LoVo cells (Supplementary Fig. 8a). However, the Snail stability positively correlates with the expression of FOXM1D in the above two cell lines after blocking protein synthesis by cycloheximide treatment (Supplementary Fig. 8b). A proteasome inhibitor MG132 treatment further supported this result (Supplementary Fig. 8c). Consistently, the results of co-IP and ICC assays showed that ectopic FOXM1D expression resulted in lower level of ubiquitin-linked Snail and higher Snail accumulation in the nucleus than the vector control in SW480 cells, while FOXM1D insufficiency induced the opposite effect in LoVo cells (Supplementary Fig. 8d, e).

Together, we identified FOXM1D as a novel modulator for class IA PI3K activity by protein-protein interaction. FOXM1D, via its exon VIII coding region, binds to the kinase domain of the catalytic p110β subunit or probable p110α/δ subunits, while via its exon II and III coding region, FOXM1D binds to the junction region between the iSH2 and cSH2 domains of the regulatory p85 subunit. This FOXM1D-mediated interaction may result in steric hindrance of the inhibitory interaction of p85 with p110, thus relieving the inhibitory effect of p85 on p110 activity and enhancing p110 activation. The subsequently potentiated PI3K/AKT activity further leads to the activation of downstream signaling proteins, which is exemplified by GSK3β-mediated Snail stabilization and nuclear translocation, thus promoting tumor EMT and metastasis (Fig. 1l).

## Supplementary information

Supplementary information

Supplementary table 1

Supplementary table 2

Supplementary information

## Data Availability

All data and materials are available to the researchers once published.
