# Supplementary material for: PI3K activation is enhanced by FOXM1D binding to p110 and p85 subunits
Source: Signal Transduct Target Ther. 2020 Jun 30;5:105. doi: 10.1038/s41392-020-00218-3 (PMC7327037; doi:10.1038/s41392-020-00218-3)
Supplement: Supplementary file 1 — Supplementary information [file 41392_2020_218_MOESM1_ESM.docx]

Supplementary Materials for

**PI3K activation is enhanced by FOXM1D binding to p110 and p85 subunits**

Qi Wang^1, 2^, Pingzhao Zhang^1, 2^, Wei Zhang^1^, Xin Zhang^1^, Jianfeng Chen^1^, Peipei Ding^1^, Luying Li^1^, Xinyue Lv^1^, Ling Li^1^, Weiguo Hu^1,^ *

^1^ Fudan University Shanghai Cancer Center and Institutes of Biomedical Sciences, Shanghai Medical College, Fudan University, Shanghai 200032, China

^2^ These authors equally contribute to the work.

*Correspondence to: Weiguo Hu ([weiguohu@fudan.edu.cn](mailto:weiguohu@fudan.edu.cn))

**This file includes:**

Materials and Methods

Fig. S1 to S8

Table S1 to S4

References

**MATERIALS AND METHODS**

**Cell Culture**

All cell lines in this study were purchased from the Type Culture Collection Cell Bank, Chinese Academy of Sciences. SW480, LoVo and 293FT cells were cultured in Dulbecco’s Modified Eagle’s Medium (HyClone, South Logan, UT，USA), HCT116 and HeLa cells were cultured in RPMI-1640 medium (HyClone) supplemented with 10% fetal bovine serum (Gibco BRL, Grand Island, NY, USA) and 1% penicillin/streptomycin (Ambion, Austin, TX, USA). All cell lines were maintained at 37℃ in a humidified incubator (Thermo Fisher Scientific, Waltham, MA, USA) with 5% CO_2_.

**Plasmid Construction**

The coding sequence (CDS) of FOXM1D was cloned^1^ and inserted into pCMV-N-FLAG, pCMV-N-HA, pGEX-6P-1, and pCDH-CMV-MCS-EF1-Puro to establish plasmids expressing FOXM1D with different tags for separate purposes. The CDSs of p110α，p110β，and p110δ were subcloned into the pCMV-N-GFP-Myc vector to express GFP-fused proteins for localization in ICC assays. FLAG-tagged p110β and Myc-tagged p85α were cloned in pCMV-N-FLAG and pCMV-N-Myc, respectively, to perform immunoprecipitation and ICC assays. The truncated mutants of FOXM1D, p110β and p85α, and the kinase domain of p110α/δ were inserted into the corresponding plasmids using specifically designed primers. Plasmids containing shRNA targeting FOXM1D (PLKO.1-puro-sh*FOXM1D*) or scrambled sequences were generated in our previous study^1^. Specific primers used are listed in Table S3.

**Lentivirus Production and Stable Cell Line Establishment**

Plasmids that overexpress FOXM1D (pCDH-CMV-MCS-EF1-Puro-FOXM1D) or silence FOXM1D (PLKO.1-puro-sh*FOXM1D*) and relevant vector plasmids were co-transfected with psPAX2 and pMD2.G into 293FT cells by Neofect™ DNA transfection reagent (Neofect Biotech, Beijing, China). Lentiviral particles were collected at 48 hour post-transfection and added to the culture medium of SW480, HeLa and LoVo cells respectively. Then, the stable cell lines were selected by puromycin (2μg/mL, Solarbio life sciences, Beijing, China).

**Plasmid and siRNA Transfection**

Plasmids and siRNAs were transfected into cells with Neofect™ DNA transfection reagent (Neofect Biotech) or Lipofectamine® 3000 (Thermo Fisher Scientific) according to the manufacturers’ protocol respectively.

**Quantitative RT-PCR (qRT-PCR)**

Total RNAs were isolated and reversely -transcribed into cDNA using Nuclezol reagent (Macherey-Nagel, Düren, Germany) and PrimeScript™ RT Master Mix (TaKaRa, Tokyo, Japan). Then qRT-PCR assays were performed on a QuantStudio™ 7 Flex Real-Time PCR System platform (Life technologies corporation, Gaithersburg, MD) using SYBR Premix Ex Taq II (TAKARA). Specific primers used are listed in Table S3.

**Immunoblot (IB) Assay**

Cell proteins were harvested by protein extraction buffer supplemented with protease inhibitors and phosphatase inhibitors (Beyotime Biotechnology, Shanghai, China) and analyzed with a standard protocol for IB. The proteins of interest in IB were visualized using X-ray film or CCD camera imaging devices ImageQuant LAS 4000 (GE Healthcare). Antibodies used are listed in Table S4. For the EGF stimulation assay, cells were plated in six-well plates and starved overnight with serum-free media. Then, the cells were stimulated with EGF (PeproTech, Rocky Hill, NJ, USA) at the indicated concentrations and times, washed by PBS for twice and then rapidly lysed with SDS loading buffer (Thermo Fisher Scientific) and boiled for analysis with IB. PI3K inhibitor, BKM120 (Selleck Chemicals, Houston, TX, USA) and IPI145 (Selleck Chemicals) were added in the medium 12 hour prior to EGF administration.

**Co-immunoprecipitation (co-IP)**

Cell proteins were prepared as described above and incubated with protein A/G-linked magnetic beads (Thermo Fisher Scientific) and specific antibodies overnight. Next, the complex was washed with protein extraction buffer and boiled with SDS-loading buffer containing β-mercaptoethanol (Amresco, Solon, OH, USA). The co-IP samples were then subjected to LC/MS, IB analysis or silver staining. Antibodies used in co-IP assays are listed in Table S4.

**GST Pull-Down Assays**

GST-tagged FOXM1D was induced to be expressed in Rosetta (DE3) cells and purified by GST resin (Merck Millipore, Billerica, MA, USA). Then, the total extracts of cells overexpressing p110β or p85α were incubated with the purified complex overnight. After several washes with PBST (0.02% Tween-20 in PBS), the protein complex was denatured with reducing SDS-loading buffer and heated at 100°C for 10 min. The IB assay was then performed to detect the relevant proteins.

**Immunocytochemistry (ICC) Staining**

Cells were planted on round coverslips which were embedded in wells of a 24 well plate overnight. The cells were then transfected by plasmids. For stable cell lines, the transfection steps were omitted. After 48 h of transfection, the cells were fixed with 4% paraformaldehyde (Yeasen Biotechnology, Shanghai, China) for 30 min at room temperature, permeabilized with 0.2% Triton X-100/PBS on ice for 8-10 min, blocked with 1% BSA/PBS and sequentially incubated with primary and secondary antibodies for 2 h at room temperature. Finally, the cells were stained with a mounting reagent containing DAPI (Yeasen Biotechnology, Shanghai, China), and the round coverslips were placed upside down on a glass slide and observed on a Leica TCS-SP5 confocal system (Leica, Wetzlar, Germany). The antibodies applied in the ICC assay are listed in Table S4. The fluorescent distribution of different protein in ICC images are analyzed by the Plot Profile of Image J and the data was presented by GraphPad Prism 7^2-6^. A line segment was chosen for analysis randomly and the software read the fluorescence value from the start point to the end point of the labelled line segment in turn. The X axis in the immunofluorescence quantification images means the relative distance from the start point of the labelled lines used for immunofluorescence quantification, and the Y axis means the intensity detected for different channels. The quantification of co-localization of ICC assays is analyzed by the Co-localization Threshold of Image J^7-9^ and presented as the scatter plot and Pearson’s correlation coefficient. In the scatter plot, the closer the scatter diagram is to the diagonal, the higher the degree of co-localization is. The value of Pearson’s correlation coefficient ranges from -1.0 to 1.0, in which 0 indicates no significant co-localization, 0-1.0 indicate different level of co-localization, and 1.0 indicates 100% co-localization rate. The scatter plot and the Pearson’s correlation coefficient is analyzed on the whole picture or a whole cell, not a random line segment.

**Transwell Migratory Assay**

Cells were harvested, resuspended with no-serum medium and seeded in the upper chamber of a transwell (Corning, Corning, NY, USA). The whole set was inserted in wells of a 24 well plate in which complete medium containing 10% FBS was added. The cells migrated to the lower chamber were fixed with 4% paraformaldehyde and stained with 0.5% crystal violet (Solarbio life sciences). The images were captured using an Olympus microscope system (Olympus, Tokyo, Japan) and the cell numbers in the images were counted.

**Protein Stability Assay**

Stable cell lines were seeded and treated with cycloheximide (20 μM, Merck Sigma-Aldrich, St. Louis, MO, USA) or MG132(10 μM, Selleck Chemicals) for indicated time and then were lysed directly using SDS loading buffer to measure the half-life and stability of Snail in IB assays. To further determine the ubiquitination level of Snail, proteins were immunoprecipitated by antibody against Snail and analyzed using ubiquitin antibody in IB assays.

**Gel Chromatography**

Proteins derived from HeLa-Vector and HeLa-FOXM1D cells were harvested, quantified and adjusted to the same concentration. Equal volumes of HeLa-Vector and HeLa-FOXM1D proteins were injected into the AKTA pure chromatography system (GE Healthcare, Little Chalfont, Buckinghamshire, UK) with a Superdex gel filtration column (Superdex 200; Amersham Biosciences, USA). The molecular mass of each tube referred to a Gel Chromatography Calibration Kit HMW (GE Healthcare). The fractions were collected to validate the existence of the polymers of FOXM1D, p110 and p85 by IB analysis.

**Statistical Analysis**

The quantitative data of the transwell and qRT-PCR assays are shown as the mean±SD and were analyzed with Student’s *t*-test (two-tailed, unpaired). *P*<0.05 was defined as significant.

**Figure. S1.**

**
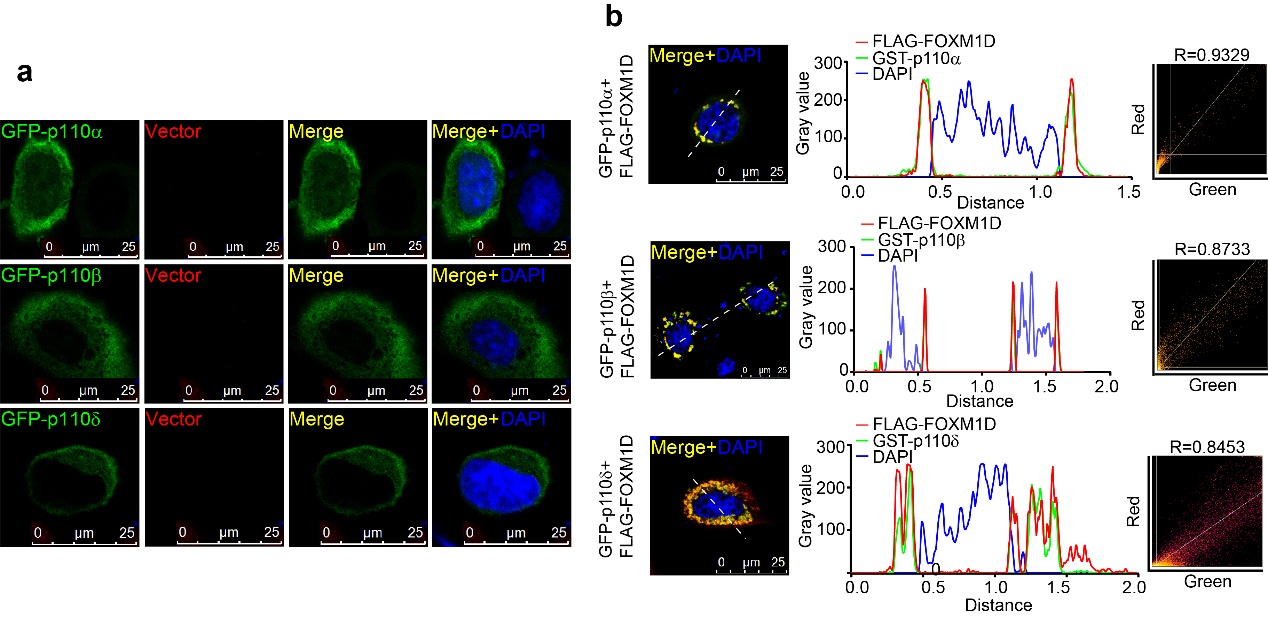
**

**Figure S1. The supplemental experiments for validating the co-localization between FOXM1D and p110 in Fig 1c.**

**a** The negative controls in ICC assays of Fig 1c. Cells transfected with the vector plasmid pCMV-C–FLAG did not show any specific fluorescence and GFP-p110 did not co-localize with “Vector” in cells. **b** The quantification data of Fig 1c. The scatter plot and the Pearson’s correlation coefficient is analyzed on the whole picture. The similar fluorescent pattern, the overlapped scatter plot, and the high Pearson’s correlation coefficient showed the co-localization between FOXM1D and p110. R: Pearson’s correlation coefficient. Scale bar, 25 μm.

Figure. S2.


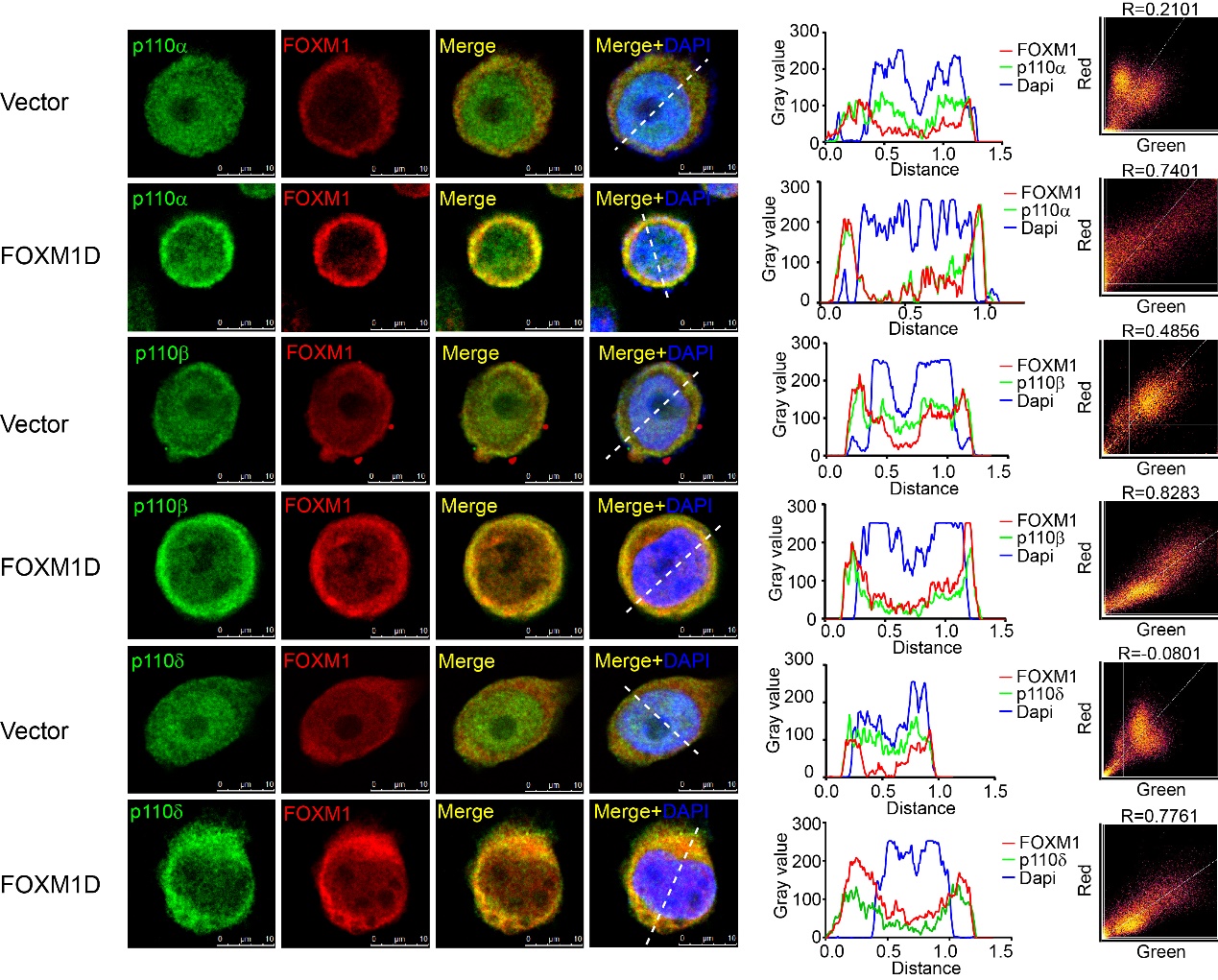


**Figure S2. Triple ICC staining for endogenous p110 (green), FOXM1 (red), and nuclei (DAPI, blue) in SW480-Vector and SW480-FOXM1D cell lines.** The quantification data on the right of each group of ICC images showed that more p110α/β co-localized with FOXM1D in SW480-FXOM1D cells than that in SW480-Vector cells. The scatter plot and the Pearson’s correlation coefficient is analyzed on the whole picture. R: Pearson’s correlation coefficient. Scale bar, 10 μm.

Figure. S3.


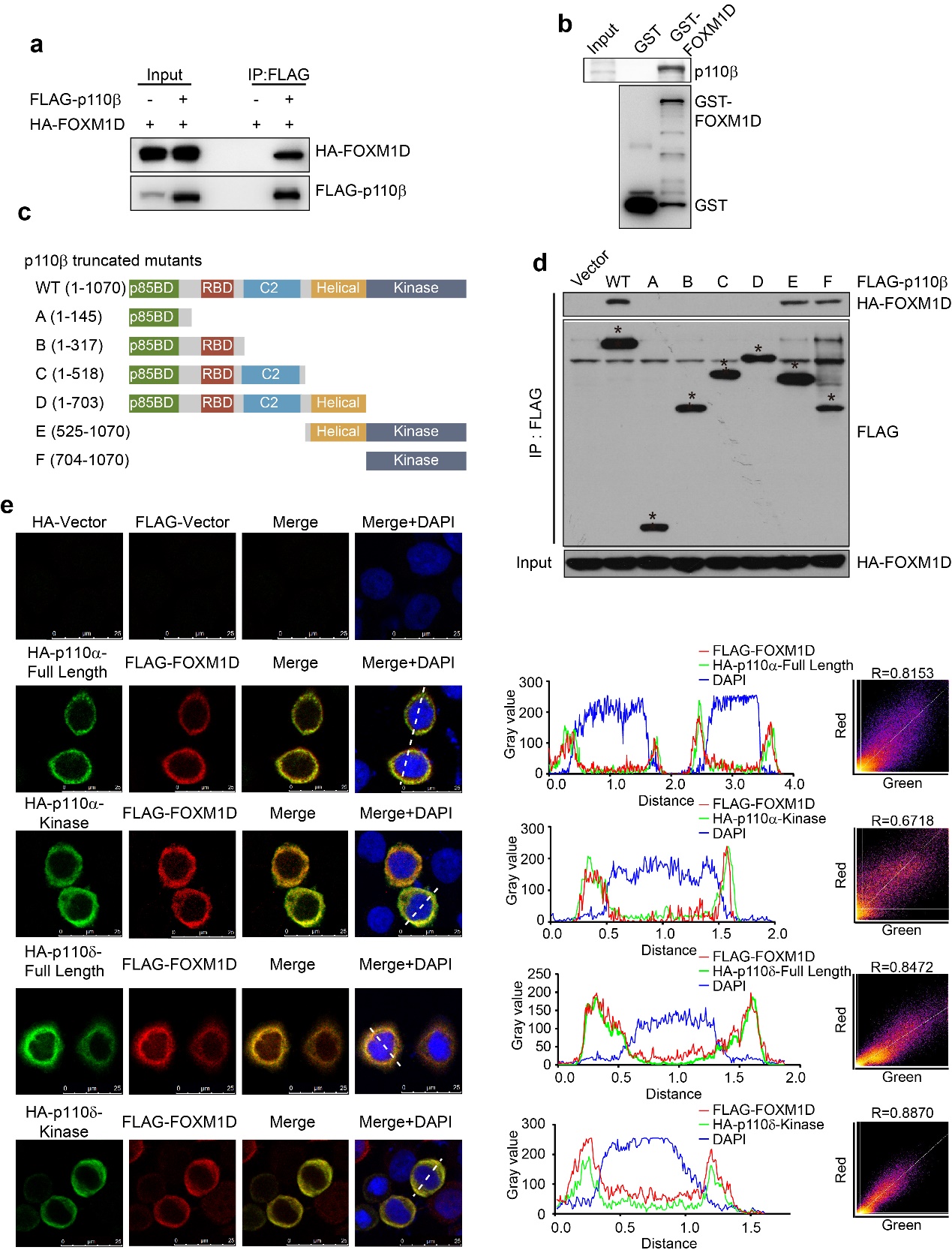


**Figure S3. FOXM1D directly interacts with the class IA p110 catalytic subunit.**

**a** IP was performed in lysates of 293FT cells expressing HA-FOXM1D and FLAG-p110β using an anti-FLAG antibody. HA-FOXM1D and FLAG-p110β were detected by IB using HA and FLAG antibodies, respectively. **b** GST-FOXM1D was incubated with extracts of 293FT cells expressing p110β, followed by IB using p110β and GST antibodies. **c, d** Identification of the mutual binding sites of FOXM1D and p110β. Co-IP assays were performed from 293FT cells co-transfected with FLAG-p110β or FLAG-tagged truncated mutants of p110β (**c**) and HA-FOXM1D expressing plasmids using a FLAG antibody (**d**). The obtained samples were detected by IB using FLAG and HA antibodies (**d**). **e** ICC assays to detect the co-localization between the kinase domains of p110α/δ and FOXM1D. The quantification data shown on the right verified the co-localization between the kinase domains of p110α/δ and FOXM1D. The scatter plot and the Pearson’s correlation coefficient is analyzed on the line-labelled cells. R: Pearson’s correlation coefficient. Scale bar, 25 μm.

Figure. S4.

**
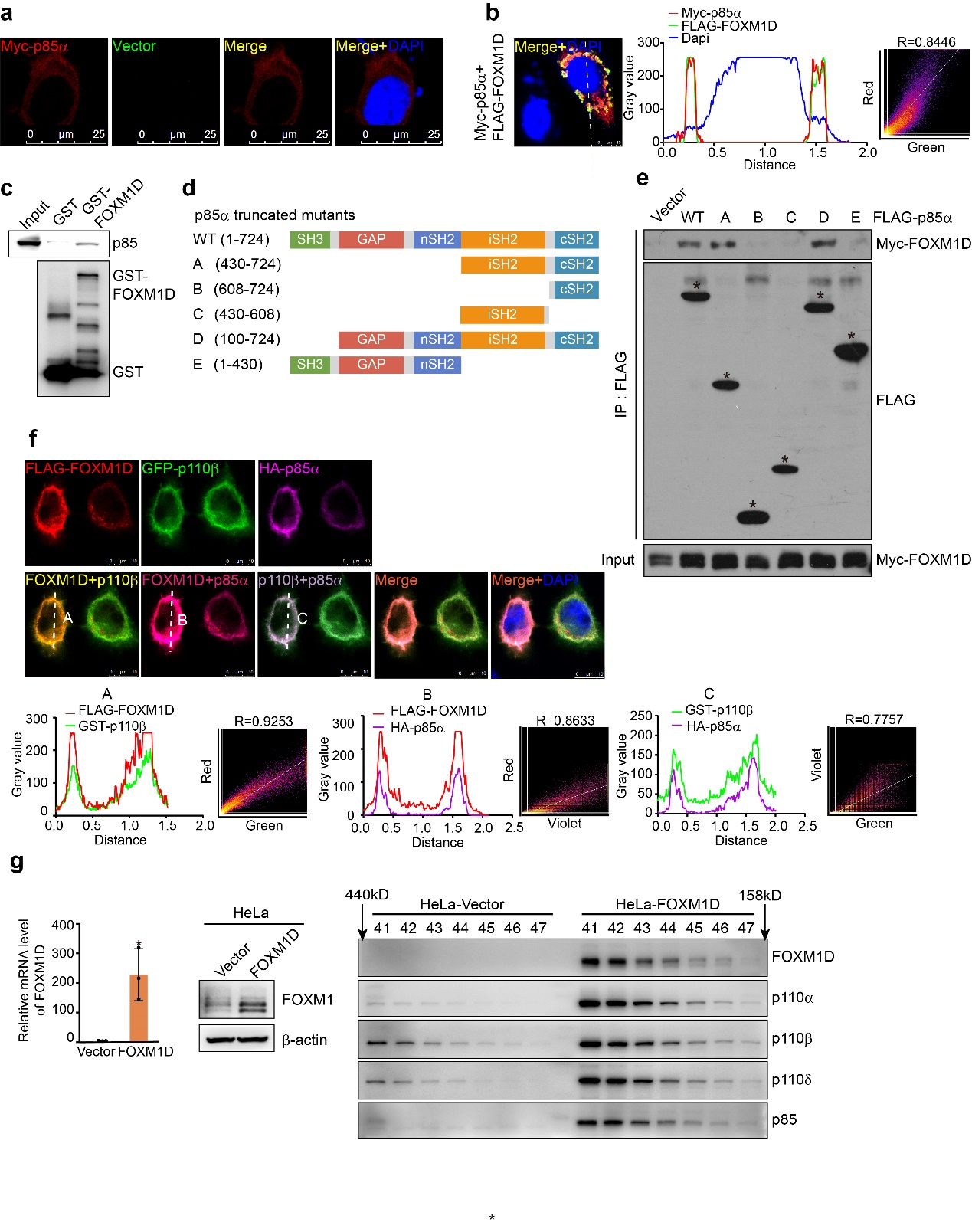
**

**Figure S4. FOXM1D directly interacts with the class IA p85α regulatory subunit.**

**a**, **b** The supplemental experiments for validating the co-localization between FOXM1D and p85 in Fig 1h. **a** The negative controls in ICC assays of Fig 1h. Scale bar, 25 μm. **b** The quantification data of Fig 1h. The similar fluorescent pattern, the overlapped scatter plot, and the high Pearson’s correlation coefficient showed the co-localization between FOXM1D and p85α. The scatter plot and the Pearson’s correlation coefficient is analyzed on the line-labelled cells. R: Pearson’s correlation coefficient. Scale bar, 10 μm. **c** GST-FOXM1D was incubated with 293FT cell extracts expressing p85α, followed by IB using p85 and GST antibodies. **d, e** Identification of the mutual binding sites of FOXM1D and p85α. Co-IP assays were performed of extracts from the 293FT cells expressing FLAG-p85α or FLAG-tagged truncated mutants of p85α (**d**) and Myc-FOXM1D-expressing plasmids using a FLAG antibody (**e**). The obtained samples were detected by IB using FLAG and Myc antibodies (**e**). **f** ICC staining for FLAG-FOXM1D (red), HA-p85α (violet), GFP-p110β (green), and nuclei (DAPI, blue) was performed in HeLa cells co-transfected with FLAG-FOXM1D-, HA-p85α-, and GFP-p110β-overexpressing plasmids. Scale bar, 10 μm. The quantification data presented below the ICC images showed the interaction between FLAG-FOXM1D and GFP-p110β, between FLAG-FOXM1D and HA-p85α, between GFP-p110β and HA-p85α at the same time in one single cell respectively. The scatter plot and the Pearson’s correlation coefficient is analyzed on the line-labelled cells. Therefore, we can conclude that FOXM1D, p110 and p85 can interact with each other and function as a protein complex. R: Pearson’s correlation coefficient. **g** The gel chromatography to determine the physiological existence of FOXM1D, p110α/β/δ and p85 protein complex in the lysates derived from HeLa cells with ectopic FOXM1 expression. Error bars: mean ± SD (n=3); Student’s t-test; *, *P*<0.05.

Figure. S5.


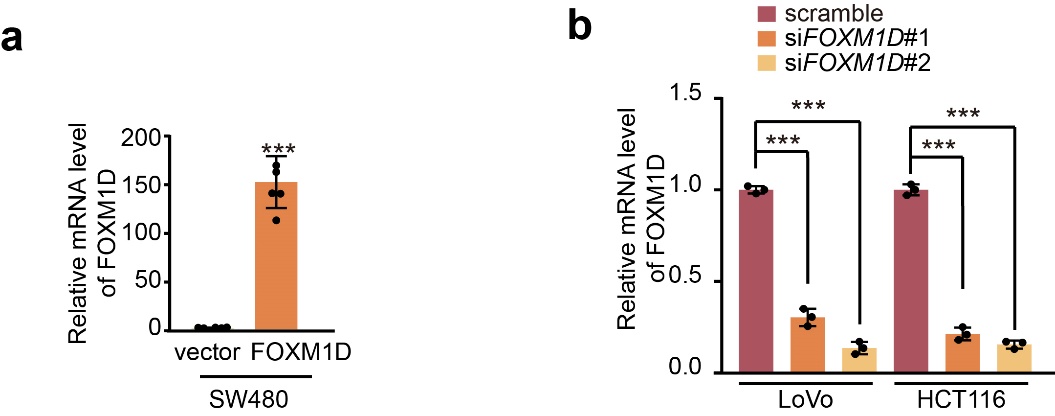


**Figure S5.** The mRNA levels of *FOXM1D* were detected by qRT–PCR in the SW480-Vectorr/FOXM1D stable cell lines (**a**) and siRNA-induced FOXM1D-decifient LoVo and HCT116 cells. Error bars: mean ± SD (n=5 for **a**, n=3 for **b**); Student’s t-test; ***, *P*<0.001.

Figure. S6.


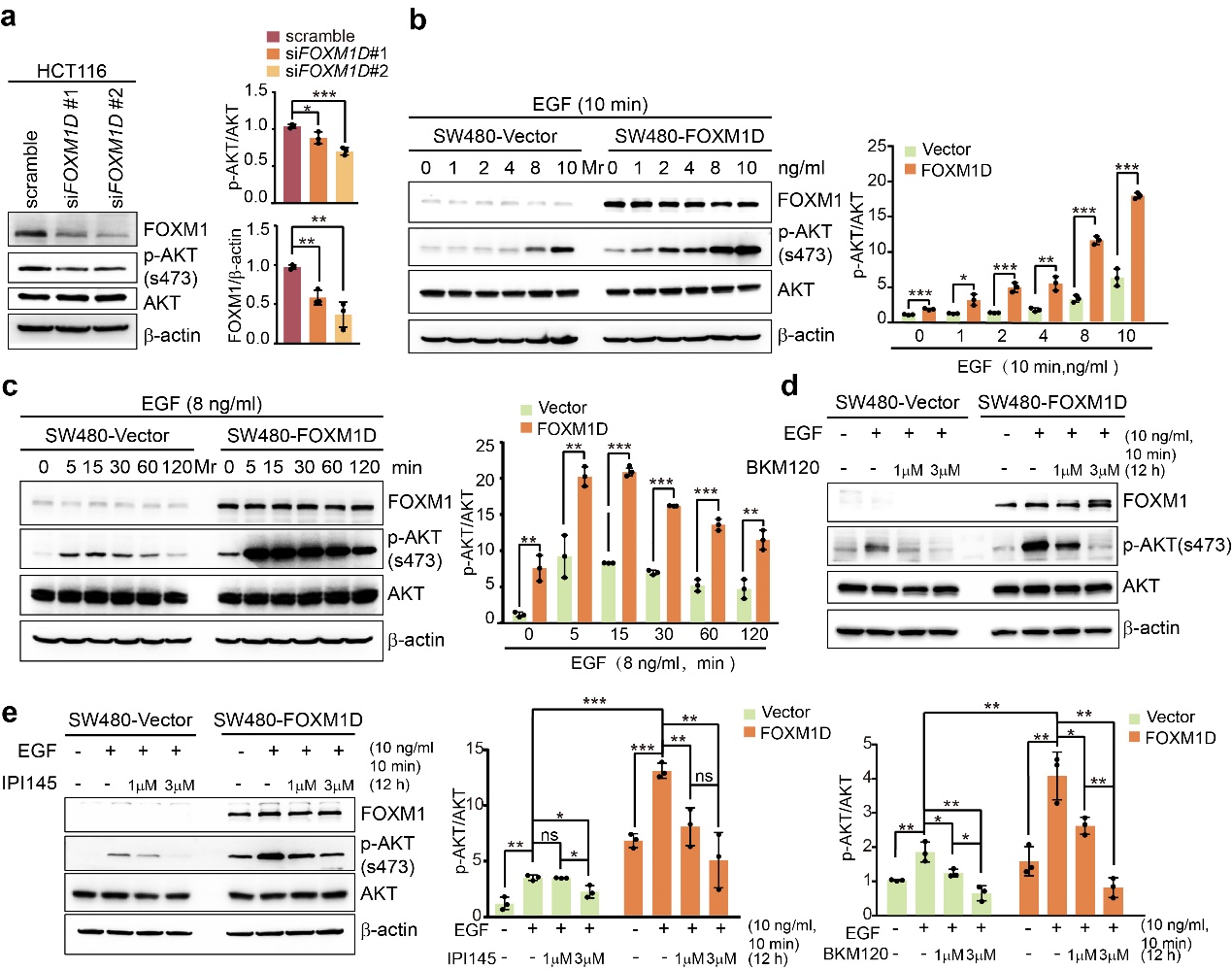


**Figure S6. FOXM1D binding to PI3K regulates PI3K activity.**

**a** HCT116 cells were transfected with siRNAs targeting FOXM1D, and the cell extracts were analyzed with IB. **b** SW480-FOXM1D and control cells were serum-starved overnight and stimulated with 0-10 ng/ml EGF for 10 min, followed by detection with IB. **c** SW480-FOXM1D and control cells were serum-starved overnight and stimulated with 8 ng/ml EGF for the indicated time periods, followed by detection with IB analysis. **d, e** SW480-FOXM1D and control cells were starved and treated with the PI3K inhibitor BKM120 (**d**) or IPI145 (**e**) for 12 h prior to stimulation with 10 ng/ml EGF for 10 min. Then, the cells were harvested and analyzed in IB. The quantitative results are presented on the right (except for that of figure **d** which is shown at the bottom). Error bars: mean ± SD (n=3); Student’s t-test; *, *P*<0.05, **, *P*<0.01 ***, *P*<0.001.

Figure. S7.


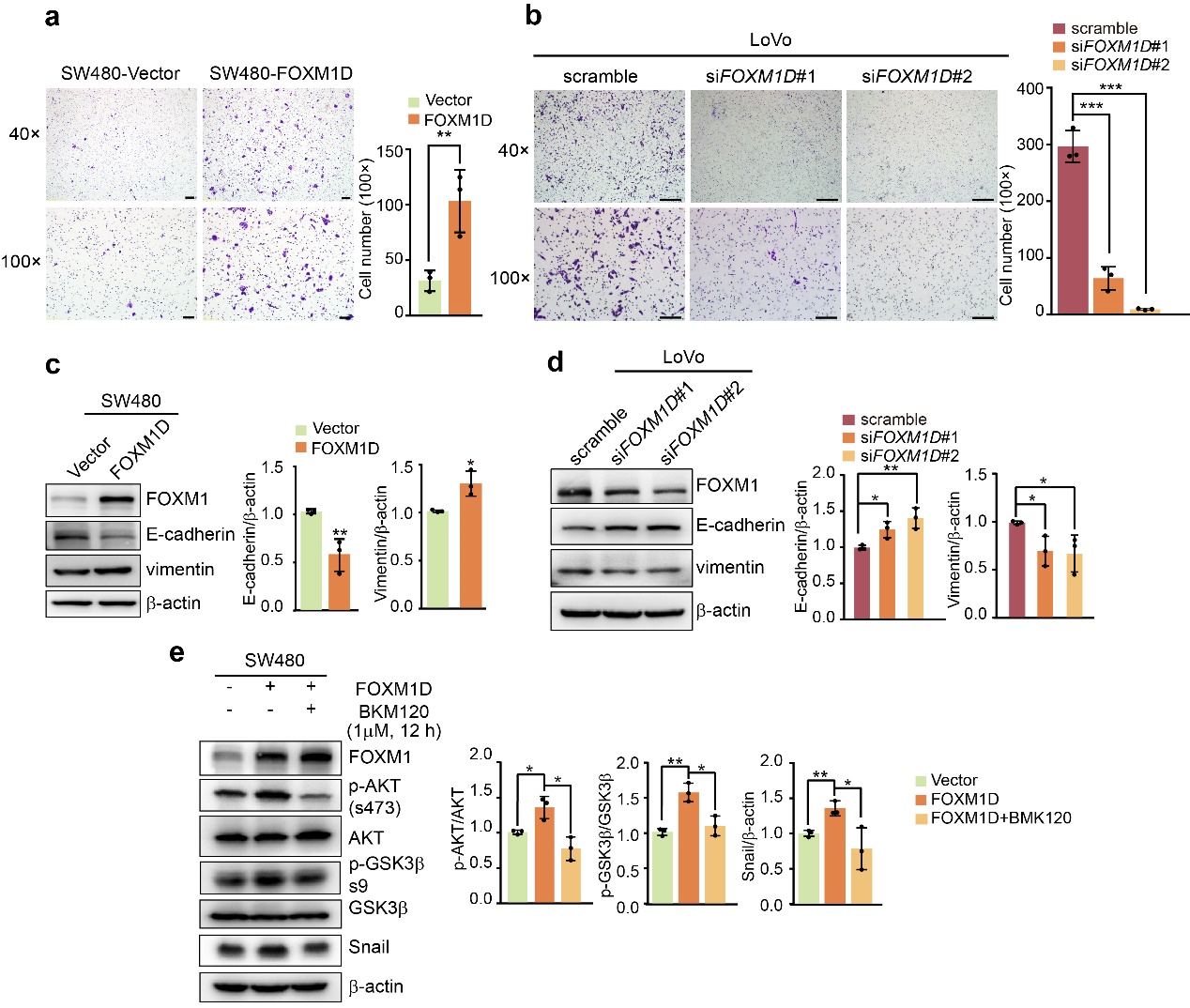


**Figure S7. FOXM1D promotes the migration of colorectal cancer cells by inducing EMT via activation of the GSK3β-Snail pathway.**

**a**, **b** The migratory abilities of the SW480-FOXM1D stable cell line (**a**) and LoVo-si*FOXM1D* cells (**b**) were detected by transwell assays. The scale bars represent 200 μm (40×) and 100 μm (100×) respectively in **a** and 500 μm (40×) and 200 μm (100×) in **b**. **c**, **d** Cell extracts of the SW480-FOXM1D stable cell lines (**c**) and LoVo-si*FOXM1D* cells (**d**) were harvested and analyzed in IB assays to evaluate the levels of EMT markers, E-cadherin and vimentin. **e** SW480 cells were transfected with the FLAG-FOXM1D and vector control plasmids and treated with the PI3K inhibitor BKM120 (1 μM). Cells were harvested 12 h post transfection and analyzed with IB using the indicated antibodies. The quantitative results are presented on the right. Error bars: mean ± SD (n=3); Student’s t-test; *, *P*<0.05, **, *P*<0.01 ***, *P*<0.001.

Figure. S8.

**
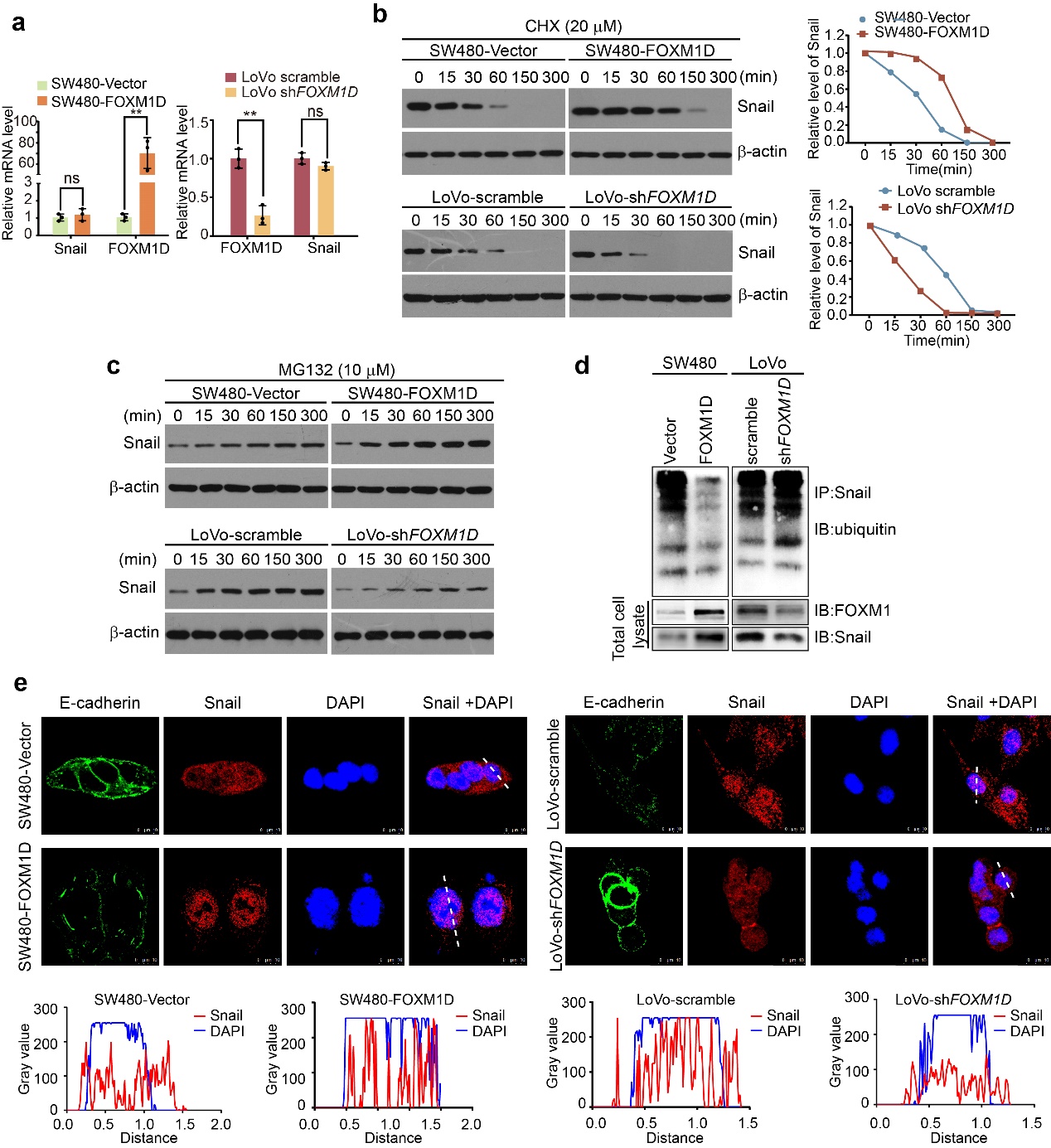
**

**Figure S8. Snail is stabilized via suppressed proteasome pathway and increased nuclear accumulation in FOXM1D-ovexpressing cells.**

**a** The mRNA levels of *FOXM1D* and *Snail* were detected by qRT–PCR in the SW480-FOXM1D and LoVo-sh*FOXM1D* cell lines. Error bars: mean ± SD (n=3); Student’s t-test; **, *P*<0.01. **b** Lysates of the indicated SW480 and LoVo stable cell lines which were treated with 20 µM cycloheximide for different time intervals were harvested and analyzed by IB. **c** Cell extracts of the indicated stable cell lines which were treated with 10 µM MG132 for different time intervals were harvested and analyzed by IB. **d** Lysates of the indicated SW480 and LoVo stable cell lines were subjected to IP analyses with a Snail antibody followed by IB. **e** The cellular location and level of Snail (red), E-cadherin (green), and nuclei (DAPI, blue) were observed in triple ICC staining in the indicated SW480 and LoVo stable cell lines. The scale bars represent 10 μm. The quantification data presented below ICC images showed that more Snail accumulated in the nuclear in SW480-FOXM1D cells and less Snail accumulated in the nuclear in LoVo-sh*FOXM1D* cells.

**Table S1 (separate file)**

Proteins identified by LC-MS in co-immunoprecipitates of Control

**Table S2 (separate file)**

Proteins identified by LC-MS in co-immunoprecipitates of FOXM1D

**Table S3. Nucleotides sequences used in different experiments**

|  | Primers for qRT-PCR |
| --- | --- |
| *FOXM1D* | F-primer: 5’-CAGGTGTTTAAGCAGCAGA-3’  R-primer: 5’-GGTGATGGGTGTACCAAAAT-3’ |
| *SNAl1* | F-primer: 5’-CCCCAATCGGAAGCCTAACT-3’  R-primer: 5’-GGACAGAGTCCCAGATGAGC-3’ |
| *ACTB(β-Actin)* | F-primer: 5’-CGGGAAATCGTGCGTGAC-3’  R-primer: 5’-CAGGGAGGAGCTGGAAGC-3’ |
|  | siRNA sequences |
| si*FOXM1D* #1 | sense: 5’-CCCAUCACCAGCUUGUUUATT-3’  antisense: 5’-UAAACAAGCUGGUGAUGGGTT-3’ |
| si*FOXM1D* #2 | sense: 5’-CAGGUGGUGUUUGGUUACATT-3’  antisense: 5’-UGUAACCAAACACCACCUGTT-3’ |
| Scrambled Control | sense: 5’-UUCUCCGAACGUGUCACGUTT -3’  antisense: 5’-ACGUGACACGUUCGGAGAATT -3’ |
|  | Primers for plasmids construction (without restriction site) |
| FOXM1D | F-primer: 5’-ATGAAAACTAGCCCCCGTC-3’  R-primer: 5’-CTACTGTAGCTCAGGAATAAACTGG-3’ |
| p110α | F-primer: 5’-ATGCCTCCACGACCATCATCAGGT-3’  R-primer: 5’-TCAGTTCAATGCATGCTGTTTAAT-3’ |
| p110δ | F-primer: 5’-ATGCCCCCTGGGGTGGACTGCC-3’  R-primer: 5’-CTACTGCCTGTTGTCTTTGGACA-3’ |
| FOXM1D(1-217) | F-primer: 5’-ATGAAAACTAGCCCCCGTCG-3’  R-primer: 5’-CTATAACCTGTCGCTGCTCCAGG-3’ |
| FOXM1D(1-348) | F-primer: 5’-ATGAAAACTAGCCCCCGTCG-3’  R-primer: 5’-CTAGTGCGCCCAGGGGGAGTTCG-3’ |
| FOXM1D(1-454) | F-primer: 5’-ATGAAAACTAGCCCCCGTCG-3’  R-primer: 5’-CTATAAACAAAGAAAGATAAAAT-3’ |
| FOXM1D(347-768) | F-primer: 5’-ATGCGGCGGAAGATGAAGCCACTG-3’  R-primer: 5’-CTACTGTAGCTCAGGAATAAACT-3’ |
| FOXM1D(403-768) | F-primer: 5’-ATGAGGTTTTTGGGGAACAGGTG-3’  R-primer: 5’-CTACTGTAGCTCAGGAATAAACT-3’ |
| FOXM1D(445-768) | F-primer: 5’-ATGCCTCAGCTAGCAGCACCTGA-3’  R-primer: 5’-CTACTGTAGCTCAGGAATAAACT-3’ |
| FOXM1D(218-348) | F-primer: 5’-ATGAAGGTTGAGGAGCCTTCGAGA-3’  R-primer: 5’-CTATGCGCCCAGGGGGAGTTCGGT-3’ |
| p110β(1-1070) | F-primer: 5’-ATGTGCTTCAGTTTCATAATGCCTCCT-3’  R-primer: 5’-TTAAGATCTGTAGTCTTTCCGAACTGTG-3’ |
| p110β(1-145) | F-primer: 5’-ATGTGCTTCAGTTTCATAATGCCTCCT-3’  R-primer: 5’-CTTATACTTCAGGATCCTTCAAGGAATC-3’ |
| p110β(1-317) | F-primer: 5’-ATGTGCTTCAGTTTCATAATGCCTCCT-3’  R-primer: 5’-TTATGGTGGTAATGGAAGAGGAAG-3’ |
| p110β(1-518) | F-primer: 5’-ATGTGCTTCAGTTTCATAATGCCTCCT-3’  R-primer: 5’-TTAACTTGACACATTAGCACTATCAC-3’ |
| p110β(1-703) | F-primer: 5’-ATGTGCTTCAGTTTCATAATGCCTCCT-3’  R-primer: 5’-TTACACACTTCCCCGGCAGTATG-3’ |
| p110β(525-1070) | F-primer: 5’-ATGCTAATGTGTCAAGTCGAGGTG-3’  R-primer: 5’-TTAAGATCTGTAGTCTTTCCGAACTGTG-3’ |
| p110β(704-1070) | F-primer: 5’-ATGTGGGGCACATGAAAGTGCTTTC-3’  R-primer: 5’-TTAAGATCTGTAGTCTTTCCGAACTGTG-3’ |
| p110β(700-850) | F-primer: 5’-ATGGCCGGGGAAGTGTGGGGCAC-3’  R-primer: 5’-TTAGAGGCCAGAGCGATCTCCTGTTG-3’ |
| p110β(775-925) | F-primer: 5’-ATGAAAAGTGCAAATACATGGATTCC-3’  R-primer: 5’-TTAGATGTTGTCACTATGTCTGTC-3’ |
| p110β(850-1000) | F-primer: 5’-ATGGCCTCATTGAAGTTGTGAGCAC-3’  R-primer: 5’-TTAGAAGAGATTCCCATGCCGTCG-3’ |
| p110β(925-1070) | F-primer: 5’-ATGACATCATGGTCAAAAAAACTGGC-3’  R-primer: 5’-TTAAGATCTGTAGTCTTTCCGAACTGTG-3’ |
| p85α(1-724) | F-primer: 5’-ATGAGTGCTGAGGGGTACCAG-3’  R-primer: 5’-TCATCGCCTCTGCTGTGCATATACTG-3’ |
| p85α(1-430) | F-primer: 5’-ATGAGTGCTGAGGGGTACCAG-3’  R-primer: 5’-TCAATACTGGATAAAGTAATTTCA-3’ |
| p85α(100-724) | F-primer: 5’-ATGCAGGTTCTTCGAAAACTGAAGC-3’  R-primer: 5’-TCATCGCCTCTGCTGTGCATATACTG-3’ |
| p85α(430-724) | F-primer: 5’-ATGCCAAATACCAACAGGATCAAG-3’  R-primer: 5’-TCATCGCCTCTGCTGTGCATATACTG-3’ |
| p85α(579-724) | F-primer: 5’-ATGACCAATACTTGATGTGGTTGACTC-3’  R-primer: 5’-TCATCGCCTCTGCTGTGCATATACTG-3’ |
| p85α(608-724) | F-primer: 5’-ATGCTGGTGGAAGATGATGAAGAT-3’  R-primer: 5’-TCATCGCCTCTGCTGTGCATATACTG-3’ |
| p85α(579-700) | F-primer: 5’-ATGACCAATACTTGATGTGGTTGACTC-3’  R-primer: 5’-TCAGTGTTGGTAATGTAGCACCAG-3’ |
| p85α(430-608) | F-primer: 5’ -ATGCCAAATACCAACAGGATCAAG-3’  R-primer: 5’-TCATGAATATTGGTCTTCAGTGTTTTC-3’ |
| p110α-Kinase (697-1069) | F-primer: 5’ -ATGTATTTGAAGCACCTGAATAG-3’  R-primer: 5’-TCAGTTCAATGCATGCTGTTTAAT-3’ |
| p110δ-Kinase (677-1045) | F-primer: 5’ -ATGCACCACATGAAGGTGCTGATGAA-3’  R-primer: 5’-CTACTGCCTGTTGTCTTTGGACA-3’ |
|  | shRNA oligonucleotide sequences |
| sh*FOXM1D* | 5’-CCGGAACAGGTGGTGTTTGGTTACACTCGAG TGTAACCAAACACCACCTGTTTTTTTG-3’  5’-AATTCAAAAAAACAGGTGGTGTTTGGTTACACT  CGAGTGTAACCAAACACCACCTGTT-3’ |
| shRNA-control | 5’-CCGGAATTCTCCGAACGTGTCACGTCTCGAGAC  GTGACACGTTCGGAGAATTTTTTTG-3’  5’-AATTCAAAAAAATTCTCCGAACGTGTCACGTCTCG  AGACGTGACACGTTCGGAGAATT-3’ |

**Table S4. The commercial antibodies used in this study.**

| **Antibodies** | **Manufacturers** | **Applications in this study** | **Catalog Number** |
| --- | --- | --- | --- |
| FOXM1(A11) | Santa Cruz Biotechnology | IB (1:500),  ICC (1:50) | sc-271746 |
| E-cadherin | Cell Signaling Technology | IB (1:1,000)  ICC (1:50) | 14472 |
| Vimentin | Abcam | IB (1:1,000) | ab92547 |
| β-actin (C4) | Santa Cruz Biotechnology | IB (1:500) | sc-47778 |
| PI3 Kinase p110α (C73F8) | Cell Signaling Technology | IB (1:1,000)  ICC (1:100) | 4249 |
| PI3 Kinase p110β (C33D4) | Cell Signaling Technology | IB (1:1,000)  ICC (1:100) | 3011 |
| PI3 Kinase p110δ (D1Q7R) | Cell Signaling Technology | IB (1:1,000)  ICC (1:100) | 34050 |
| PI3 Kinase p85 (19H8) | Cell Signaling Technology | IB (1:1,000) | 4257 |
| Phospho-Akt (Ser473) (D9E) | Cell Signaling Technology | IB (1:1,000) | 4060 |
| Akt | Proteintech | IB (1:1,000) | 10176-2-AP |
| DDDDK-tag | MBL | IB (1:1,000)  IP (2ug/test)  ICC (1:500) | M185 |
| GST (26H1) | Cell Signaling Technology | IB (1:1,000) | 2624 |
| HA-Tag (TANA2) | MBL | IB (1:1,000)  ICC (1:500) | M180 |
| Myc-Tag (9B11) | Cell Signaling Technology | IB (1:1,000)  ICC (1:200) | 2276 |
| Phospho-GSK-3β (Ser9) | Cell Signaling Technology | IB (1:1,000) | 3011 |
| GSK-3β (D5C5Z) | Cell Signaling Technology | IB (1:1,000) | 12456 |
| Snail (C15D3) | Cell Signaling Technology | IB (1:1,000)  IP (2ug/test) | 3879 |
| Snail | Proteintech | ICC (1:200) | 13099-1-AP |
| Ubiquitin (P4D1) | Cell Signaling Technology | IB (1:1,000) | 3936 |

**REFERENCES**

1 Zhang, X. *et al.* A novel FOXM1 isoform, FOXM1D, promotes epithelial-mesenchymal transition and metastasis through ROCKs activation in colorectal cancer. *Oncogene* **36**, 807-819, doi:10.1038/onc.2016.249 (2017).

2 Fuchsova, B., Novak, P., Kafkova, J. & Hozak, P. Nuclear DNA helicase II is recruited to IFN-alpha-activated transcription sites at PML nuclear bodies. *The Journal of cell biology* **158**, 463-473, doi:10.1083/jcb.200202035 (2002).

3 Park, L. *et al.* Brain Perivascular Macrophages Initiate the Neurovascular Dysfunction of Alzheimer Abeta Peptides. *Circulation research* **121**, 258-269, doi:10.1161/circresaha.117.311054 (2017).

4 Farias, G. G., Guardia, C. M., Britt, D. J., Guo, X. & Bonifacino, J. S. Sorting of Dendritic and Axonal Vesicles at the Pre-axonal Exclusion Zone. *Cell reports* **13**, 1221-1232, doi:10.1016/j.celrep.2015.09.074 (2015).

5 Hui, E. *et al.* T cell costimulatory receptor CD28 is a primary target for PD-1-mediated inhibition. *Science (New York, N.Y.)* **355**, 1428-1433, doi:10.1126/science.aaf1292 (2017).

6 Yokosuka, T. *et al.* Programmed cell death 1 forms negative costimulatory microclusters that directly inhibit T cell receptor signaling by recruiting phosphatase SHP2. *The Journal of experimental medicine* **209**, 1201-1217, doi:10.1084/jem.20112741 (2012).

7 Dunn, K. W., Kamocka, M. M. & McDonald, J. H. A practical guide to evaluating colocalization in biological microscopy. *Am J Physiol Cell Physiol* **300**, C723-742, doi:10.1152/ajpcell.00462.2010 (2011).

8 Bolte, S. & Cordelières, F. P. A guided tour into subcellular colocalization analysis in light microscopy. *Journal of microscopy* **224**, 213-232, doi:10.1111/j.1365-2818.2006.01706.x (2006).

9 Zinchuk, V., Wu, Y. & Grossenbacher-Zinchuk, O. Bridging the gap between qualitative and quantitative colocalization results in fluorescence microscopy studies. *Sci Rep* **3**, 1365, doi:10.1038/srep01365 (2013).
